# Internal Parameters Calibration of Vision Sensor and Application of High Precision Integrated Detection in Intelligent Welding Based on Plane Fitting

**DOI:** 10.3390/s22062117

**Published:** 2022-03-09

**Authors:** Chuanhui Zhu, Zhiming Zhu, Zhijie Ke, Tianyi Zhang

**Affiliations:** Key Laboratory for Advanced Materials Processing Technology, Ministry of Education, Department of Mechanical Engineering, Tsinghua University, Beijing 100084, China; zhuch20@mails.tsinghua.edu.cn (C.Z.); kzj17@tsinghua.org.cn (Z.K.); zhangty14@tsinghua.org.cn (T.Z.)

**Keywords:** vision sensing, internal parameters integrated calibration, plane fitting, welding groove sizes, relative position and posture

## Abstract

Vision sensing is a key technology to realize on-line detection of welding groove sizes and welding torch relative position and posture parameters during the arc welding process of intelligent production. For the specially designed vision sensor based on combined laser structured lights, an integrated calibration method for its internal parameters is proposed firstly, which improves the efficiency, accuracy and comprehensiveness of internal parameter calibration for a line structured light vision sensor and provides a good foundation for industrial application of the vision sensor. Then, the high precision integrated detection algorithms are derived for the V-groove size parameters and the spatial position and posture (SPP) parameters of the welding torch relative to the welding groove based on a single modulated laser lines image. The algorithms make full use of the data in a single modulated laser lines image, adopting data segmentation and plane fitting to realize the 3D reconstruction of V-groove surfaces and its adjacent workpiece surfaces of planar workpiece, so solving the parameters with high precision. In the verification tests, the relative detection error of V-groove size parameters of planar workpiece is less than 1%, and the relative detection error of SPP parameters of welding torch relative to the welding groove is less than 5%, which separately shows the effectiveness and accuracy of the calibration method and the detection algorithms. This research work provides a good technical support for the practical application of the specially designed vision sensor in the intelligent welding production.

## 1. Introduction

For the welding of workpieces with determined groove shape and size located in specified spatial posture, the spatial position and posture (SPP) of the welding torch relative to the welding groove have great influence on weld formation quality that cannot be ignored. Thus, they should be reasonably set prior to implementing the arc welding process in addition to the basic process parameters such as welding current, arc voltage, welding speed and motion trajectory of the welding torch. However, machining and assembly errors for the welding groove sizes usually exist, and thermal deformation when workpieces are being welded inevitably occurs. This will often lead to defects and deficiencies with the weld formation if only relying on the preset SPP as well as the motion trajectory of the welding torch relative to the welding groove. Thus, it is necessary during the actual welding procedure to carry out high-precision welding groove sizes detection and weld tracking, that is, the motion trajectory control of the welding torch and synchronous control on the SPP of the welding torch relative to the welding groove. In other words, this important direction in the welding field is worthy of research and development to realize intelligent welding by on-line sensing detection and real-time feedback control, which can not only improve the efficiency of welding production but also effectively ensure the quality of the welded joint and product [1,2].

Vision sensing is a key technology to realize intelligent welding. Compared with other vision sensing technologies, the line structured light vision sensing based on the perspective projection principle has the comprehensive advantages of simple operation, high detection accuracy and low system cost and has been widely used in modern industrial production [3].

The premise of a vision sensor based on line structured light successfully applied in realizing high precision detection is firstly to calibrate its internal parameters effectively, including camera calibration and structured light plane calibration [4]. In the camera calibration, Zhang’s camera calibration method [5] is flexible and convenient and has been widely used. On the calibration of the structured light plane, there are many studies in recent years. Some specially designed 3D targets were used as calibrators [6,7,8], and good calibration results of the structured light plane were obtained. However, these 3D calibration methods rely on high precision 3D targets or motion devices, which greatly limit their application. Then, some planar calibration methods have been developed. Xu et al. [9] constructed the laser structured light plane through multiple Plücker matrices of the 3D crossing lines between the target planes and the laser projection planes. Ha [10] established 3D–3D correspondences between the camera and laser range finder by a calibration structure that has a triangular hole on its plane. Chen et al. [11] proposed a geometric calibration method for a line structured light sensor, which calibrates camera intrinsic parameters and the laser structured light plane equation by using a single circular target designed to construct geometric constraints. However, these calibration methods of the structured light plane need a specially designed target, auxiliary equipment or a relatively complex calibration model, which reduce their applicability and convenience.

Meanwhile, the line structured light vision sensor has been widely used in the welding field, such as weld tracking, welding torch location determining and so on [12,13,14]. However, the intelligent welding also requires on-line detection of the SPP parameters of the welding torch relative to the welding groove, reasonably adjusting and controlling them according to the actual shape and size parameters detected in the welding groove, so as to obtain high quality weld formation.

In terms of the welding groove size parameters detection based on line structured light vision sensing, some typical research works are as follows. He et al. [15] and Zhu et al. [16] realized the detection of weld geometry sizes, including width, height, etc., by using a corresponding image processing algorithm. Kim et al. [17] proposed a point cloud registration technique for multiple weld seams, which realized the 3D information extraction of weld seams. However, when using the vision sensor based on single line structured light for the welding groove size parameters detection, it is necessary to preset the height and posture of the sensor relative to the measured workpiece and for them to remain unchanged during the welding process, otherwise the vision sensor needs to be recalibrated. To solve this problem, Guo et al. [18,19] proposed a new vision sensor based on combined laser structured lights and derived the corresponding detection algorithm, which realizes the detection of welding groove size parameters and position deviation of the welding torch relative to the welding groove. However, this detection algorithm is based on the condition that the vision sensor is perpendicular to the workpiece surface when it is applied for the detection of planar workpiece, which still has certain limitations.

In terms of the relative position and posture detection based on line structured light vision sensing, some research works have also been carried out. Xue et al. [20] and Zeng et al. [21] used a crosshair laser projected onto the workpiece surface and obtained the position and posture between the welding torch and the groove by the combination of 2D and 3D information in the laser images. Kiddee et al. [12] obtained the relative position and posture of edges of a V-groove weld seam by using a modified template matching for ROI image set by the cross mark of the structured light. Zhang [22] proposed a mathematical model of SPP detection of the welding torch relative to the welding groove based on a combined laser structured lights vision sensor and realized the detection of position and angle parameters of the welding torch relative to the welding groove.

From the above brief review and analysis, we can see that the current calibration method of the line structured light vision sensor is complex and inconvenient. Meanwhile, in the detection process, the mentioned detection methods for the welding groove sizes and welding torch relative position and posture parameters depend on sensor installation and welding equipment motion parameters at different degrees. There are few studies that have completed the integrated detection of welding groove sizes and welding torch relative position and posture parameters with high detection accuracy, robustness and adaptability at the same time.

Aiming at the existing problems mentioned above, on the basis of the deduced detection mathematical model of the vision sensor based on combined laser structured lights, this paper firstly proposes an integrated calibration method using only an ordinary checkerboard calibration board for internal parameters of the vision sensor, including camera internal parameters (*f_x_*, *f_y_*, *u*_0_, *v*_0_, *k*_1_, *k*_2_) and the structured light plane equation parameters (*A_l_*_1_, *B_l_*_1_, *C_l_*_1_, *D_l_*_1_ and *A_l_*_2_, *B_l_*_2_, *C_l_*_2_, *D_l_*_2_) in a camera coordinate system. Next, based on the processing of single modulated laser lines image captured by camera, the 3D point cloud data of the laser lines can be obtained. Then, the V-groove surfaces and its adjacent workpiece surfaces of the planar workpiece are reconstructed by data segmentation and plane fitting. Finally, the integrated detection of the V-groove size parameters (groove width *b*_1_, *b*_2_ and groove depth *h*) of the planar workpiece and the relative position and posture parameters (position parameters *e*, γ, *H* and posture parameters *α*, *β*) of the welding torch in any relative position and posture is realized. This research work effectively improves the robustness and applicability of detection for the vision sensor and has important application value in intelligent welding production.

## 2. Configuration and Detection Mathematical Model of Vision Sensor

### 2.1. Configuration of Vision Sensor Based on Combined Laser Structured Lights

**Figure 1:**
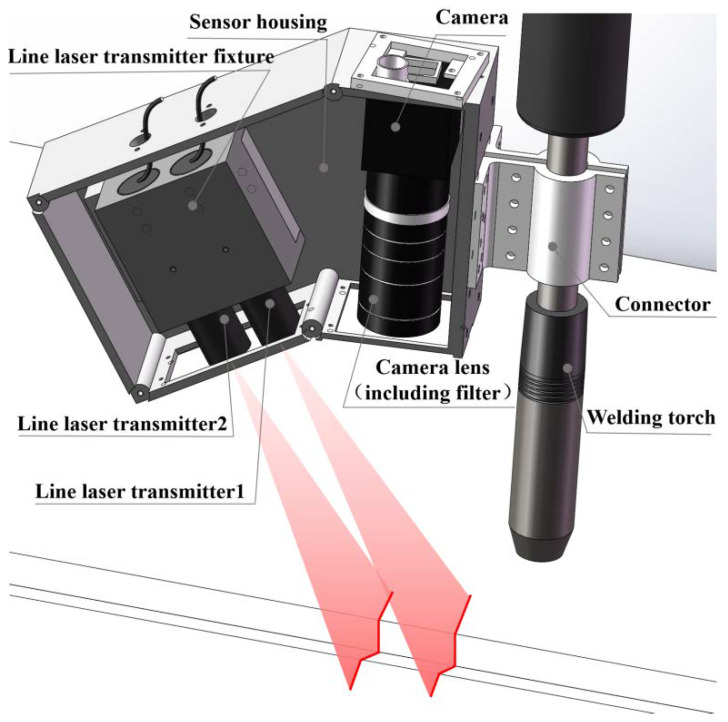
Schematic diagram of vision sensor based on combined laser structured lights.

The application of the single line structured light vision sensor in the market needs to preset and keep its fixed installation position and posture when detecting the sizes of the object, and it needs multiple scans to obtain the accurate sizes of the object [23]. Meanwhile, a binocular or multilocular vision sensor also has problems processing multiple images at the same time, complex calibration and so on. Thus, a vision sensor based on combined laser structured lights is designed (as shown in Figure 1) and the corresponding detection algorithm is proposed, which can realize the detection of welding groove sizes and the welding torch relative position and posture parameters under any relative position and posture, and has great detection applicability, robustness and accuracy.

The vision sensor shown in Figure 1 is mainly composed of a monocular camera and two line laser transmitters. It adopts the spatial arrangement of oblique incidence-perpendicular receiving, which has the advantages of small detection error and compact structure [24,25]. Among them, the central axes of the two line laser transmitters are parallel, and the designed angle value between the optical axis of the camera and the central axes of the line laser transmitters is 30°, which makes the detected values of the vision sensor close to the actual values [26]. The vision sensor is fixedly mounted on the forward side of the welding torch (welding direction). Meanwhile, the central axis of the welding torch, the optical axis of the camera and the central axes of two line laser transmitters are theoretically coplanar; this plane should be perpendicular to the width direction of the camera image plane theoretically. In addition, based on the spectral characteristics that the arc light intensity of GMAW is the weakest within the wavelength range of 620–700 nm, the wavelength of laser transmitters selected is 660 nm; a narrow-band filter with the wavelength of 660 ± 8 nm is selected and installed in front of the camera lens to filter out the arc interference and ensure high laser light transmittance. The selected main components and their parameters of the vision sensor are shown in Table 1.

### 2.2. Detection Mathematical Model of Vision Sensor

The two line laser transmitters of the vision sensor (Figure 1) emit two parallel light planes with a certain thickness and project onto the surfaces of the measured object (workpiece and welding groove). Then, two laser lines modulated by the welding groove are formed on the surfaces of the measured object. The modulated laser lines are captured by the CMOS camera, and the 2D coordinates of the laser lines can be extracted by processing a single image. After that, according to the internal parameters of the vision sensor and the coordinate transformation based on the perspective projection principle, the 3D coordinates of the points in the modulated laser lines projected on the surfaces of the measured object in the camera coordinate system can be solved. Then, the welding groove sizes and the SPP parameters of the welding torch relative to the welding groove can be obtained.

**Figure 2:**
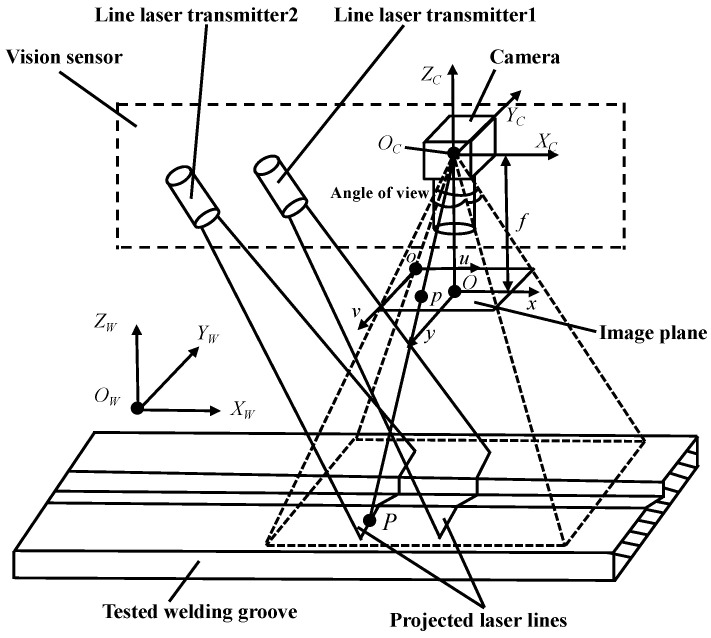
Schematic diagram of working principle of vision sensor.

In order to convert the 2D image data of the modulated laser lines captured by the camera into 3D space data through the perspective projection model, four rectangular coordinate systems are established, as shown in Figure 2. In Figure 2, *O*-*xy* is a 2D image coordinate system (taking the intersection *O* of the camera optical axis *O_C_Z_C_* and the image plane as the origin) and *o*-*uv* is a 2D pixel coordinate system, which are used to characterize the 2D coordinates in the image plane; *O_C_*-*X_C_Y_C_Z_C_* is the 3D camera coordinate system (*O_C_* is the optical center of the camera) and *O_W_*-*X_W_Y_W_Z_W_* is the 3D world coordinate system (*f* is the focal length of the camera), which are used to characterize the 3D coordinates of space points in the actual physical space. Point *P* is the sampling point in the modulated laser lines projected on the surfaces of the measured object and point *p* is the perspective imaging point of the sampling point *P* on the 2D image plane.

In Figure 2, the coordinate transformation of the sampling point *P* from the 3D world coordinate system *O_W_-X_W_Y_W_Z_W_* to the 3D camera coordinate system *O_C_-X_C_Y_C_Z_C_* is a position and posture transformation of rigid body (including rotation and translation). This transformation matrix is called the external parameter matrix of the camera and it can be expressed as follows [27]:

(1) $$\left[\begin{array}{c} X_{C}\\ Y_{C}\\ Z_{C}\\ 1 \end{array}\right]=\left[\begin{array}{cc} R_{C} & T\\ \vec{0} & 1 \end{array}\right]\left[\begin{array}{c} X_{W}\\ Y_{W}\\ Z_{W}\\ 1 \end{array}\right]$$

where *R_C_* is a 3 × 3 matrix and *T* is a 3 × 1 matrix, which represent the posture and position of the camera coordinate system *O_C_-X_C_Y_C_Z_C_* in the world coordinate system *O_W_-X_W_Y_W_Z_W_*, respectively.

According to the perspective projection principle, the matrix relationship between the coordinates of the sampling point *P* in the 3D camera coordinate system *O_C_-X_C_Y_C_Z_C_* and the coordinates of the imaging point *p* in the 2D image coordinate system *O*-*xy* is as follows:

(2) $$Z_{C}\left[\begin{array}{c} x\\ y\\ 1 \end{array}\right]=\left[\begin{array}{cccc} \begin{array}{l} f\\ 0\\ 0 \end{array} & \begin{array}{l} 0\\ f\\ 0 \end{array} & \begin{array}{l} 0\\ 0\\ 1 \end{array} & \begin{array}{l} 0\\ 0\\ 0 \end{array} \end{array}\right]\left[\begin{array}{c} X_{C}\\ Y_{C}\\ Z_{C}\\ 1 \end{array}\right]$$

On the image plane, the pixel widths along the *x* and *y* directions are d*x* and d*y* (mm/pixel), respectively, and *u*_0_, *v*_0_ (pixel) are the position coordinates of the origin of the 2D image coordinate system *O*-*xy* in the 2D pixel coordinate system *o*-*uv*. Then, the matrix relationship of the imaging point *p* between its image coordinates (*x*, *y*) and pixel coordinates (*u*, *v*) is:

(3) $$\left[\begin{array}{c} u\\ v\\ 1 \end{array}\right]=\left[\begin{array}{ccc} \frac{1}{\mathrm{dx}} & 0 & u_{0}\\ 0 & \frac{1}{\mathrm{dy}} & v_{0}\\ 0 & 0 & 1 \end{array}\right]\left[\begin{array}{c} x\\ y\\ 1 \end{array}\right]$$

Ignoring the camera imaging distortion, the sampling point *P*, the imaging point *p* and the camera optical center *O_C_* meet geometric collinear constraint. According to the conversion matrix of Equations (1)–(3), the conversion relationship between the 3D world coordinates of sampling point *P* and the 2D pixel coordinates of imaging point *p* can be obtained:

(4) $$Z_{C}\left[\begin{array}{c} u\\ v\\ 1 \end{array}\right]=\left[\begin{array}{cccc} f_{x} & 0 & u_{0} & 0\\ 0 & f_{y} & v_{0} & 0\\ 0 & 0 & 1 & 0 \end{array}\right]\left[\begin{array}{cc} R_{c} & T\\ \vec{0} & 1 \end{array}\right]\left[\begin{array}{c} X_{W}\\ Y_{W}\\ Z_{W}\\ 1 \end{array}\right]$$

where *f*_x_ = *f*/d*x* and *f*_y_ = *f*/d*y* represent the dimensionless scale factor of the camera in the *x* direction and *y* direction, respectively.

However, the camera lens always has radial distortion and tangential distortion. In general, the tangential distortion is small and can be ignored, and only the radial distortion needs to be considered. The mathematical model used frequently for removing radial distortion of the camera is the Brown model [28], which is described by Taylor series expansion around the main point of image. Generally, only the first two items need to be used. The radial distortion correction formula is:

(5) $$\left[\begin{array}{c} x_{c}\\ y_{c} \end{array}\right]=(1{+k}_{1}r^{2}{+k}_{2}r^{4})\left[\begin{array}{c} x\\ y \end{array}\right]$$

where *k*_1_ and *k*_2_ represent the radial distortion coefficient of the camera, *r* represents the normalized distance between the distortion point and the main point of the image and *x_c_* and *y_c_* represent the image coordinates after radial distortion correction. So, *k*_1_, *k*_2_, *f_x_*, *f_y_*, *u*_0_ and *v*_0_ are called internal parameters of the camera system.

Only relying on the constraint provided by Equation (4), the detection mathematical model of the vision sensor is incomplete. Considering that the sampling point *P* on the surfaces of the measured object is also a point on the structured light plane projected by the line laser transmitter, a complete vision sensing detection mathematical model can be established by taking the structured light plane equation in the 3D camera coordinate system *O_C_-X_C_Y_C_Z_C_* as a supplementary condition. The general expression of the laser structured light plane equation in the 3D camera coordinate system *O_C_-X_C_Y_C_Z_C_* is:

(6) $$A_{l}X_{C}{+B}_{l}Y_{C}{+C}_{l}Z_{C}{+D}_{l}=0$$

where *A_l_*, *B_l_*, *C_l_* and *D_l_* are the coefficients of the laser structured light plane equation.

Combining the above equations, the detection mathematical model of the vision sensor based on combined laser structured lights are obtained, as shown in Equation (7). Through this equation, the 3D coordinates (*X*_C_, *Y*_C_, *Z*_C_) of the sampling point *P* in the 3D camera coordinate system *O_C_-X_C_Y_C_Z_C_* can be solved by the pixel coordinates (*u*, *v*) of the imaging point *p* in the image plane.

(7) $$\left\{ \begin{array}{l} Z_{C}\left[\begin{array}{c} u\\ v\\ 1 \end{array}\right]=\left[\begin{array}{cccc} f_{x} & 0 & u_{0} & 0\\ 0 & f_{y} & v_{0} & 0\\ 0 & 0 & 1 & 0 \end{array}\right]\left[\begin{array}{c} X_{C}\\ Y_{C}\\ Z_{C}\\ 1 \end{array}\right]\\ A_{l}{}_{1}X_{C}{+B}_{l}{}_{1}Y_{C}{+C}_{l}{}_{1}Z_{C}{+D}_{l}{}_{1}=0\mathrm{or}A_{l}{}_{2}X_{C}{+B}_{l}{}_{2}Y_{C}{+C}_{l}{}_{2}Z_{C}{+D}_{l}{}_{2}=0 \end{array}\right.$$

where *A_l_*_1_, *B_l_*_1_, *C_l_*_1_, *D_l_*_1_ and *A_l_*_2_, *B_l_*_2_, *C_l_*_2_, *D_l_*_2_ are the plane equation parameters of the structured light planes projected by the line laser transmitters 1 and 2 in the 3D camera coordinate system *O_C_-X_C_Y_C_Z_C_*.

## 3. Integrated Calibration for Internal Parameters of Vision Sensor

**Figure 3:**
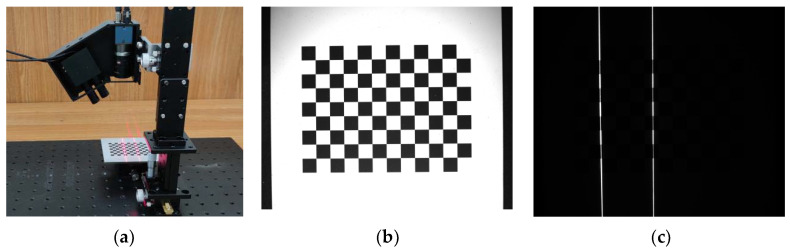
Image acquisition process of vision sensor calibration: (**a**) image acquisition, (**b**) captured image of checkerboard calibration board and (**c**) captured image of two laser projection lines.

For the vision sensor based on combined laser structured lights, an integrated calibration method is proposed and applied for the calibration of vision sensor internal parameters, including the camera internal parameters (*f_x_*, *f_y_*, *u*_0_, *v*_0_, *k*_1_, *k*_2_) and the structured light plane equation parameters (*A_l_*_1_, *B_l_*_1_, *C_l_*_1_, *D_l_*_1_ and *A_l_*_2_, *B_l_*_2_, *C_l_*_2_, *D_l_*_2_) of two line laser transmitters in the camera coordinate system. This method is only based on the ordinary planar checkerboard calibration board (its number of squares is 12 × 9, size of chessboard is 6 × 6 mm and accuracy is 1 μm), and the images of the calibration board and the laser lines are collected before and after the two line laser transmitters project the laser lines onto the calibration board by using different exposure times, respectively, as shown in Figure 3. Then, the position and posture of the calibration board are changed, and 20 sets of images of the calibration board and laser lines under different positions and postures of the calibration board are collected successively.

According to the 20 calibration board images and Zhang’s camera calibration method (see Reference [5]), the camera internal parameters can be calibrated by using the camera calibration toolbox in MATLAB. Meanwhile, the 20 sets of external parameter matrices *R_C_* and *T* of the chessboard calibration board relative to the 3D camera coordinate system *O_C_-X_C_Y_C_Z_C_* under different positions and postures can be obtained.

For the calibration of laser structured light plane equation parameters, its detailed steps are as follows. Firstly, the laser lines image is preprocessed by median filtering, binary segmentation and morphological processing. Next, the laser centerlines in the image are extracted by skeleton thinning and Hough line transform. Then, according to the obtained external parameter matrices *R*_C_, *T* of the chessboard calibration board relative to the 3D camera coordinate system *O_C_-X_C_Y_C_Z_C_* under different positions and postures and Equation (4), the 20 sets of 2D coordinate data of the laser centerlines in the laser lines image are converted into the 3D coordinate data of their corresponding point in the laser lines projected on the calibration board in the 3D camera coordinate system *O_C_-X_C_Y_C_Z_C_*. Finally, according to the 20 sets of 3D coordinate data of laser lines projected on the calibration board under different positions and postures, the two laser structured light plane equation parameters can be obtained by fitting the two structured light planes separately, which are the actual structured light planes projected from the two line laser transmitters.

The calibration results of the internal parameters for the vision sensor are shown in Table 2.

## 4. Detection Algorithm of Welding Groove Sizes and Relative Position and Posture of Welding Torch

The mathematical algorithms of vision detection are different for vision sensors with different arrangement structures between camera and laser structured light. For the designed vision sensor based on combined laser structured lights, the 3D reconstruction method for the V-groove surfaces and its adjacent workpiece surfaces of planar workpiece is primarily studied after the single modulated laser lines image processing effectively. Then, the detection algorithms of the V-groove size parameters and the SPP parameters of the welding torch relative to the welding groove are deduced. Finally, some experiments are carried out for the verification of the effectiveness and accuracy of the deduced detection algorithms.

### 4.1. Image Processing of Modulated Laser Lines Projected on V-Groove of Planar Workpiece

**Figure 4:**
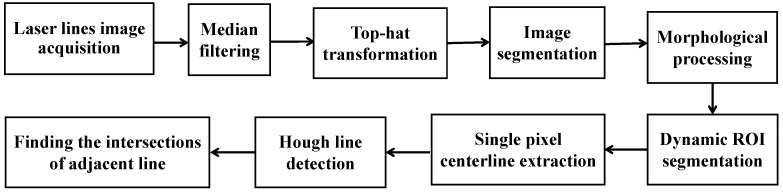
Image processing flow of modulated laser lines image.

The laser lines projected on the V-groove surfaces and its adjacent workpiece surfaces are modulated, and then the CMOS camera captures the modulated laser lines image. The flow of processing and feature extraction of modulated laser lines image is shown in Figure 4.

**Figure 5:**
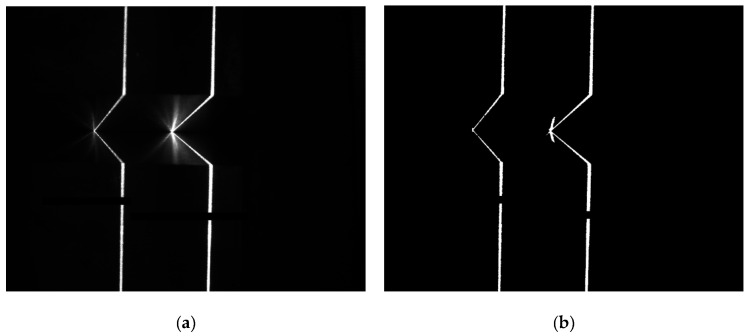

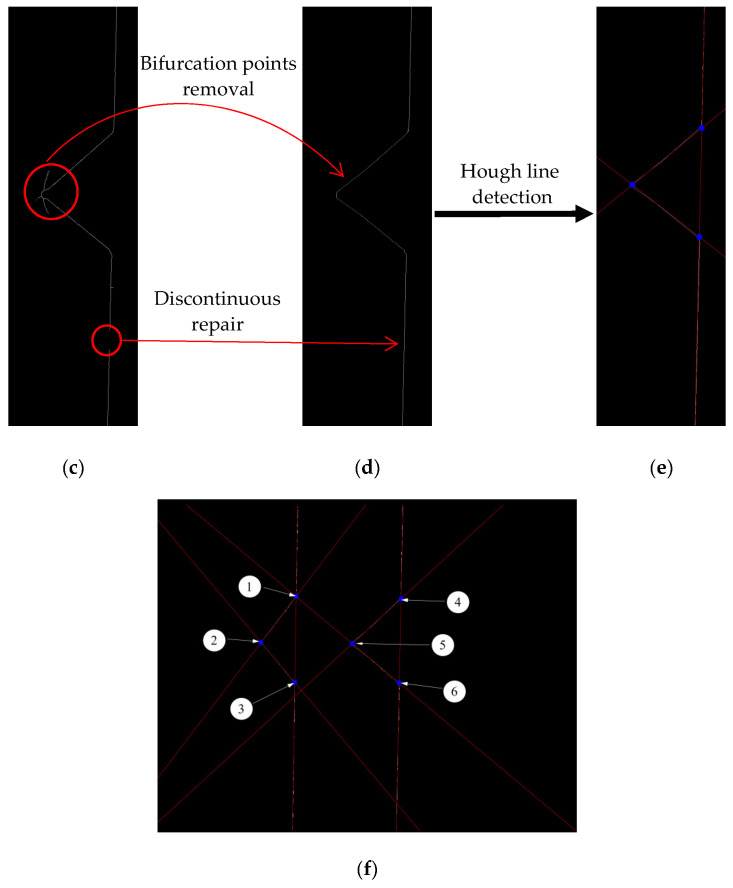
Laser lines image processing and feature extraction: (**a**) original laser lines image, (**b**) preprocessing for laser lines image, (**c**) skeleton thinning processing for ROI, (**d**) defect repair for ROI of single pixel laser line, (**e**) Hough line detection for ROI of single pixel laser line and (**f**) feature extraction of laser lines image.

Firstly, the modulated laser lines image (Figure 5a) is preprocessed by median filtering, top-hat transformation, binary segmentation and morphological processing to make the image with uniform brightness and obvious characteristics, as shown in Figure 5b. Next, according to the horizontal and vertical gray projection values of the laser lines image, the preprocessed laser lines image is segmented by the method of dynamic region of interest (ROI), and the two laser lines in the original image are divided into two small sub-region images. Extracting the features of two segmented laser line sub-images, respectively, can improve the efficiency of image processing. The features extraction of modulated laser lines includes single pixel centerline extraction, straight line fitting for centerline and intersection points calculation of adjacent fitting lines.

Considering the requirements of real-time detection accuracy and anti-interference ability of image processing, the Zhang-Suen thinning algorithm [29] is selected to process the two modulated laser line sub-images after region segmentation. The image processed by skeleton thinning may have defects such as bifurcation and discontinuity (Figure 5c). Through defect repair operations, e.g., bifurcation points removal and interpolation between discontinuous pixels, a continuous single pixel laser centerline image can be obtained (Figure 5d).

Then, for the obtained single pixel laser centerline image, the probabilistic Hough transform method is applied to determine the lines in the image. In the actual detection, the detection results of multi-group points of line segment will appear. The final detected values of the laser centerline equations can be obtained by calculating the average values of *ρ* and *θ* in polar coordinates for each line segment.

Finally, the intersection points of the two adjacent lines detected can be calculated, which are the image feature points of the V-groove of the planar workpiece (Figure 5e). For the two laser lines projected on the workpiece surfaces of V-groove, the whole laser lines image captured by camera has six feature points (Figure 5f).

### 4.2. Three-Dimensional Reconstruction for V-Groove Surfaces and Its Adjacent Workpiece Surfaces of Planar Workpiece

The obtained intersections of the fitting lines after image processing and feature extraction, which are the characteristic points of the modulated laser lines image, will inevitably have pixel errors. If the size parameters of the V-groove are directly solved by using image coordinates of these points, it will inevitably produce large detection deviation, reduce the robustness of the detection algorithm and decrease the utilization of the laser lines image data.

**Figure 6:**
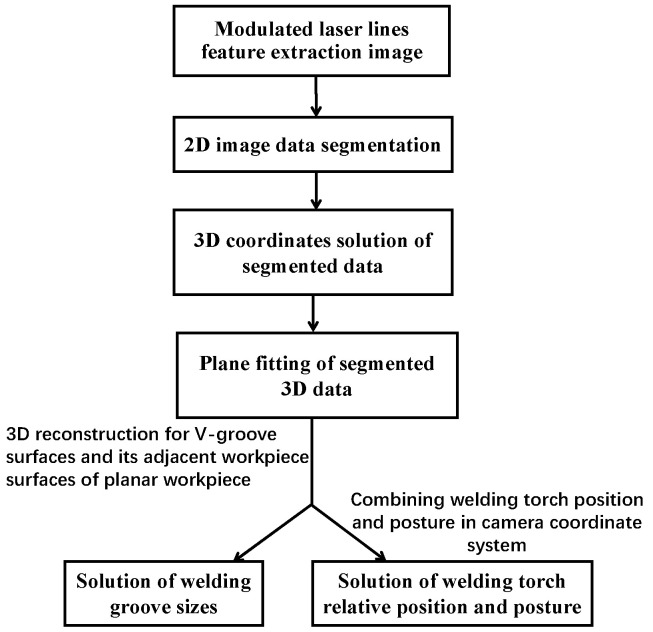
The solution flow of the welding groove sizes and welding torch relative position and posture parameters.

In order to improve the accuracy and robustness of the detection results of welding groove size parameters, a plane fitting method is proposed, that is, to reconstruct the V-groove surfaces and its adjacent workpiece surfaces based on the 3D data points of the modulated laser lines in the camera coordinate system *O_C_*-*X_C_Y_C_Z_C_*, and then to realize the high precision calculation of the welding groove sizes and welding torch relative position and posture parameters. The flow of algorithm for solving the welding groove sizes and welding torch relative position and posture parameters is shown in Figure 6.

#### 4.2.1. Two-Dimensional Image Data Segmentation of the Modulated Laser Lines

**Figure 7:**
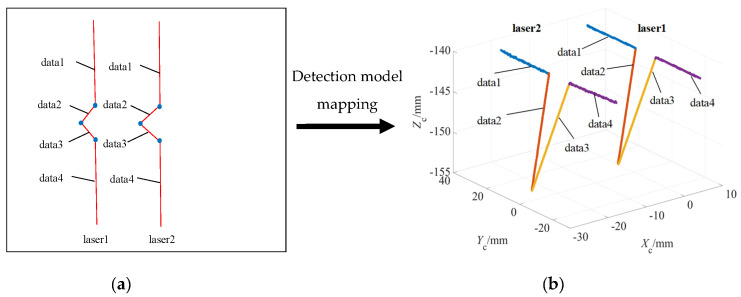
Data segmentation of modulated laser lines: (**a**) 2D data segmentation of laser lines image and (**b**) 3D data segmentation of laser lines.

The coordinates of the six feature points in Figure 5f are recorded as (*x_i_*, *y_i_*; *i* = 1, 2,..., 6) according to their serial number. Each modulated laser line has three image feature points. Taking them as breakpoints, the data of each modulated laser line are divided into four segments, which are recorded as data*i* (*i* = 1, 2, 3, 4), as shown in Figure 7a.

#### 4.2.2. Three-Dimensional Coordinates Solution of Segmented Data in the Camera Coordinate System

According to the detection mathematical model of Equation (7), the 2D segmented data of two single pixel modulated laser lines in the image coordinate system *o*-*xy* are mapped into the camera coordinate system *O_C_-X_C_Y_C_Z_C_* based on the perspective projection principle. Then, the 3D point cloud segmented data of two single pixel modulated laser lines can be obtained, as shown in Figure 7b.

#### 4.2.3. Plane Fitting of Segmented 3D Data

According to the structural characteristics of V-groove of planar workpiece, the data points with the same serial number in the two modulated laser lines are on the same welding groove surfaces or its adjacent workpiece surfaces. Theoretically, the welding groove surfaces and its adjacent workpiece surfaces are plane, so the 3D point cloud segmented data can be used for plane fitting. Four planes *S_i_* (*i* = 1, 2, 3, 4) can be obtained by fitting the data*i* (*i* = 1, 2, 3, 4) points with the same serial number in the two modulated laser lines, respectively.

Considering that the obtained six image feature points may have pixel errors, their corresponding 3D feature points are not completely accurate segment interval points. Therefore, to ensure the accuracy of plane fitting data points and reduce the influence of the data error caused by segment interval points, the segment interval points and their neighborhood data points are removed during the process of data segmentation.

The singular value decomposition (SVD) is used to plane fitting for the different segmented 3D data points separately. The coefficient matrix *M* and column matrix *X* can be constructed as follows:

(8) $$M=\left[\begin{array}{ccc} X_{C1}-{\bar{X}}_{C} & Y_{C1}-{\bar{Y}}_{C} & Z_{C1}-{\bar{Z}}_{C}\\ X_{C2}-{\bar{X}}_{C} & Y_{C2}-{\bar{Y}}_{C} & Z_{C2}-{\bar{Z}}_{C}\\ \ldots & \ldots & \ldots \\ X_{\mathrm{Ci}}-{\bar{X}}_{C} & Y_{\mathrm{Ci}}-{\bar{Y}}_{C} & Z_{\mathrm{Ci}}-{\bar{Z}}_{C} \end{array}\right],\;X=\left[\begin{array}{c} A\\ B\\ C \end{array}\right]$$

where ($\bar{X}$*_C_*, $\bar{Y}$*_C_*, $\bar{Z}$*_C_*) is the average coordinates of one 3D segmented data point (*X_Ci_*, *Y_Ci_*, *Z_Ci_*) which is used to fit corresponding plane, and column matrix X represents the normal vector of the plane.

The purpose of plane fitting is to minimize the sum of the distances between the fitting plane and all fitting points; thus, the objective function is established as follows:

(9) f(X)=min‖MX‖

The constraint condition is ‖X‖=0. In the solution process, the matrix *M* is decomposed by the method of SVD. It can be obtained that the eigenvector corresponding to the minimum singular value of the coefficient matrix *M* is the optimal solution *X* of the objective function in Equation (9). According to *D* = −(*A*$\bar{X}$*_C_* + *B*$\bar{Y}$*_C_*, + *C*$\bar{Z}$*_C_*), all of the parameter values of the fitting plane are obtained. Taking the equation parameters of four fitting planes *S_i_* as *A_i_*, *B_i_*, *C_i_* and *D_i_* (*i* = 1, 2, 3, 4), the fitting plane equations of the four planes *S*_1_, *S*_2_, *S*_3_ and *S*_4_ associated with the V-groove are:

(10) $$\left\{ \begin{array}{l} S_{1}:A_{1}X_{C}{+B}_{1}Y_{C}{+C}_{1}Z_{C}{+D}_{1}=0\\ S_{2}:A_{2}X_{C}{+B}_{2}Y_{C}{+C}_{2}Z_{C}{+D}_{2}=0\\ S_{3}:A_{3}X_{C}{+B}_{3}Y_{C}{+C}_{3}Z_{C}{+D}_{3}=0\\ S_{4}:A_{4}X_{C}{+B}_{4}Y_{C}{+C}_{4}Z_{C}{+D}_{4}=0 \end{array}\right.$$

where the normal vectors of the four planes are *m*_1_ (*A*_1_, *B*_1_, *C*_1_), *m*_2_ (*A*_2_, *B*_2_, *C*_2_), *m*_3_ (*A*_3_, *B*_3_, *C*_3_), *m*_4_ (*A*_4_, *B*_4_, *C*_4_), respectively.

**Figure 8:**
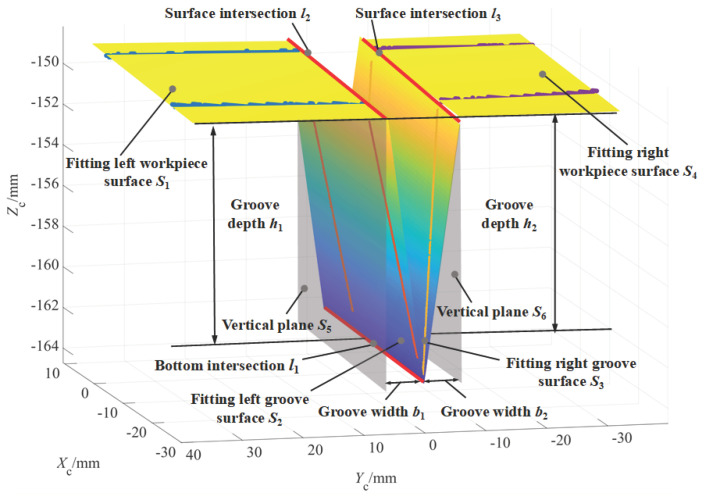
Three-dimensional reconstruction of welding groove surfaces and its adjacent workpiece surfaces of planar workpiece based on plane fitting.

The welding groove surfaces and its adjacent workpiece surfaces obtained by fitting the segmented 3D point cloud data are shown in Figure 8. So far, the 3D reconstruction of V-groove for planar workpiece is realized.

Further, according to the reconstructed 3D welding groove and the position and posture of the welding torch in the camera coordinate system *O_C_-X_C_Y_C_Z_C_*, the welding groove size parameters and the SPP parameters of the welding torch relative to the welding groove can be detected and solved.

### 4.3. Detection Algorithm of Welding Groove Size Parameters

The main size parameters of V-groove of planar workpiece include groove depth *h*, groove width *b*_1_ and *b*_2_. Their detection algorithms are as follows.

#### 4.3.1. Groove Depth h

The distance from the groove bottom intersection line *l*_1_, which is the intersection line of the left groove surface *S*_2_ and the right groove surface *S*_3_, to the left workpiece surface *S*_1_ or the right workpiece surface *S*_4_ is called the groove depth.

The linear equation of the intersection line *l*_1_ is:

(11) $$l_{1}:\left\{ \begin{array}{l} A_{2}X_{C}{+B}_{2}Y_{C}{+C}_{2}Z_{C}{+D}_{2}=0\\ A_{3}X_{C}{+B}_{3}Y_{C}{+C}_{3}Z_{C}{+D}_{3}=0 \end{array}\right.$$

The average distance from the points on the intersection line *l*_1_ to the left workpiece surface *S*_1_ and the right workpiece surface *S*_4_ are recorded as the groove depth *h*_1_ and the groove depth *h*_2_, respectively; The difference between *h*_1_ and *h*_2_ reflects the groove misalignment caused by planar workpiece assembly or thermal deformation. When the value of misalignment is small enough or negligible, take the average value of *h*_1_ and *h*_2_ as the groove depth *h*, as shown in Figure 8.

#### 4.3.2. Groove Width b_1_ and b_2_

According to the fitted left workpiece surface *S*_1_ and left groove surface *S*_2_, the intersection line *l*_2_ of the two planes can be obtained, and its linear equation is:

(12) $$l_{2}:\left\{ \begin{array}{l} A_{1}X_{C}{+B}_{1}Y_{C}{+C}_{1}Z_{C}{+D}_{1}=0\\ A_{2}X_{C}{+B}_{2}Y_{C}{+C}_{2}Z_{C}{+D}_{2}=0 \end{array}\right.$$

The direction vector ***n***_2_ of line *l*_2_ is:

***n*_2_** = ***m***_1_ × ***m***_2_ = (*B*_1_*C*_2_ − *B*_2_*C*_1_, *A*_2_*C*_1_ − *A*_1_*C*_2_, *A*_1_*B*_2_ − *A*_2_*B*_1_)

According to the direction vector ***n*_2_** of the line *l*_2_ and the normal vector ***m***_1_ of the fitting left workpiece surface *S*_1_, the normal vector ***m***_5_ of the virtual vertical plane *S*_5_, which is perpendicular to the left workpiece surface *S*_1_ and passing through the line *l*_2_, can be obtained, expressed as:

***m***_5_ = ***n*_2_** × ***m***_1_ = (*B*_1_*A*_1_*B*_2_ − *B*_1_^2^*A*_2_ − *C*_1_^2^*A*_2_ + *C*_1_*A*_1_*C*_2_, *C*_1_*B*_1_*C*_2_ − *C*_1_^2^*B*_2_ − *A*_1_^2^*B*_2_ + *A*_1_*A*_2_*B*_1_, *A*_1_*A*_2_*C*_1_ − *A*_1_^2^*C*_2_ − *B*_1_^2^*C*_2_ + *B*_1_*B*_2_*C*_1_)

Take any point of line *l*_2_, the plane equation of *S*_5_ can be obtained:

(13) $$A_{5}X_{C}{+B}_{5}Y_{C}{+C}_{5}Z_{C}{+D}_{5}=0$$

where ***m***_5_ = (*A*_5_, *B*_5_, *C*_5_), *D*_5_ = −(*A*_5_*X_C_*_q_ + *B*_5_*Y_C_*_q_ + *C*_5_Z*_C_*_q_). (*X_C_*_q_, *Y_C_*_q_, Z*_C_*_q_) are the coordinates of any point on the line *l*_2_.

Similarly, the plane equation of the virtual vertical plane *S*_6_ can also be obtained. Thus, the average distance from the points on the intersection line *l*_1_ to the virtual vertical planes *S*_5_ and *S*_6_ are the groove width *b*_1_ and *b*_2_, respectively, and the sum of *b*_1_ and *b*_2_ is the total groove width *B*, as shown in Figure 8.

### 4.4. Detection Algorithm of Welding Torch Relative SPP Parameters

#### 4.4.1. Relative SPP Parameters of Welding Torch

**Figure 9:**
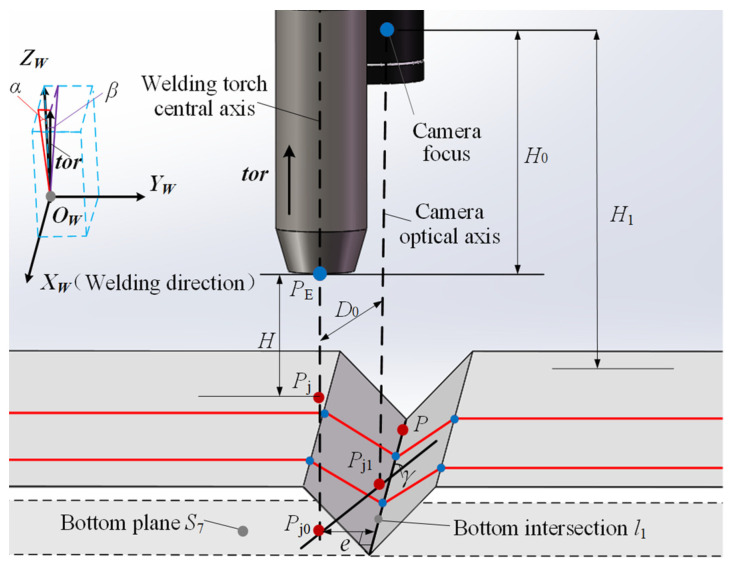
SPP of welding torch relative to welding groove.

Figure 9 is the schematic diagram of the SPP parameters of the welding torch relative to the welding groove, where *O_W_-X_W_Y_W_Z_W_* is the world coordinate system fixedly connected with the planar workpiece, *X_W_* is the welding direction (groove direction) vector, *Y_W_* is the welding groove width direction vector and *Z_W_* is the normal vector of the upper surface of the planar workpiece. The vector ***tor*** is the direction vector of the central axis of welding torch, which is used to characterize the posture of welding torch.

The relative position parameters of welding torch include transverse deviation *e*, angular deviation *γ* and welding torch height *H*.

Construct the groove bottom plane *S*_7_, which is a plane passing through the groove bottom intersection line *l*_1_ and parallel to the upper surface of the planar welded workpiece. The point *P*_j0_ and point *P*_j1_ are the intersections of the central axis of welding torch and the optical axis of CMOS camera with the bottom plane *S*_7_, respectively. The transverse deviation *e* of welding torch is defined as the distance from point *P*_j0_ to the groove bottom intersection line *l*_1_, and the angular deviation *γ* is the included angle between the line *P*_j0_*P*_j1_ and the intersection line *l*_1_.

The welding torch height *H*, camera height *H*_1_ are, respectively, the distances from the end of welding torch conductive nozzle *P*_E_, the camera focus along their respective axes to the upper surface of planar welded workpiece. The sensor installation height *H*_0_ is the distance from the camera focus along the central axis of welding torch to the end of welding torch conductive nozzle *P*_E_, which is a fixed value after the sensor being installed. When the central axis of welding torch is perpendicular to the upper surface of planar welded workpiece, the relationship among them is:

(14) $$H=H_{1}-H_{0}$$

The relative posture parameters of welding torch include front and rear tilt angle *α*, left and right tilt angle *β.* The front and rear tilt angle *α* is the included angle between the welding torch direction vector ***tor*** and the normal vector *Z_W_* of planar workpiece upper surface along the welding direction. The left and right tilt angle *β* is the included angle between the welding torch direction vector ***tor*** and the normal vector *Z_W_* of planar workpiece upper surface in the plane of groove cross section.

#### 4.4.2. Solution of Relative Position Parameters of Welding Torch

The moving direction of welding torch during arc welding process may not always coincide with the direction of welding groove, which often leads to an angular deviation *γ*. When welding process for different welding beads (layers) of V-groove are being implemented, the transverse deviation *e* and the height *H* of welding torch also need to be adjusted appropriately.

According to the plane equations of the welding groove surfaces and its adjacent workpiece surfaces and the definition of position parameters of welding torch relative to the welding groove, the transverse deviation *e,* angular deviation *γ* and welding torch height *H* are solved as follows:

1. Transverse deviation e

(1). Solve the plane equation of the bottom plane *S*_7_.

When the misalignment of the welding groove can be ignored, the average value of the normal vectors of planar workpiece upper surfaces *S*_1_ and *S*_4_ is taken as the value of the normal vector ***m***_7_ (*A*_7_, *B*_7_, *C*_7_) of the bottom plane *S*_7_, which are given as follows:

*A*_7_ = (*A*_1_ + *A*_4_)/2, *B*_7_ = (*B*_1_ + *B*_4_)/2, *C*_7_ = (*C*_1_ + *C*_4_)/2

Take any point *P* of the intersection line *l*_1_, the plane equation of the groove bottom plane *S*_7_ can be determined as:

(15) $$A_{7}X_{C}{+B}_{7}Y_{C}{+C}_{7}Z_{C}{+D}_{7}=0$$

where *D*_7_ = −(*A*_7_*X_C_*_p_ + *B*_7_*Y_C_*_p_ + *C*_7_Z*_C_*_p_), and (*X_C_*_p_, *Y_C_*_p_, *Z_C_*_p_) are the coordinates of any point *P* on the intersection line *l*_1_.

(2). Find the intersection point *P*_j0_ of the central axis of welding torch and the plane *S*_7_.

The linear equation of the central axis of welding torch is: *X_C_* = *D*_0_, *Y_C_* = 0. where *D*_0_ is the distance between the central axis of welding torch and the optical axis of camera, which are two parallel lines, and *D*_0_ is a fixed value after the sensor has been installed. The coordinates of the intersection point *P*_j0_ of the welding torch central axis and plane *S*_7_ can be solved as (*D*_0_, 0, −(*A*_7_*D*_0_ + *D*_7_)/*C*_7_).

(3). Calculate the transverse deviation *e*.

The value of the direction vector ***n***_1_ of the groove bottom intersection line *l*_1_ is:

***n*_1_** =***m***_3_ × ***m***_4_
***=*** (*B*_3_*C*_4_−*B*_4_*C*_3_, *A*_4_*C*_3_−*A*_3_*C*_4_, *A*_3_*B*_4_−*A*_4_*B*_3_)

The distance from point *P*_j0_ to intersection *l*_1_ is the transverse deviation *e*, which is:

(16) $$e=\frac{\left|PP_{j0}\times n_{1}\right|}{\left|n_{1}\right|}$$

2. Angular deviation γ

(1). Find the intersection point *P*_j1_ between the camera optical axis and plane *S*_7_.

The linear equation of the camera optical axis is: *X*_C_ = 0, *Y*_C_ = 0. The coordinates of the intersection point *P*_j1_ of the camera optical axis and plane *S*_7_ can be obtained, which are (0, 0, −*D*_7_/*C*_7_).

(2). Calculate the angular deviation *γ*.

Since the straight line *P*_j0_*P*_j1_ and the intersection line *l*_1_ are both in plane *S*_7_, their included angle is the angular deviation *γ,* which is:

(17) $$\gamma =\arccos\frac{P_{j0}P_{j1}\;•\;n_{1}}{\left|P_{j0}P_{j1}\right|\left|n_{1}\right|}$$

3. Welding torch height *H*

The coordinates of the intersection point *P*_j_ of the central axis of welding torch and plane *S*_1_ can be solved by combining two corresponding equations, which are (*D*_0_, 0, −(*A*_1_*D*_0_ + *D*_1_)/*C*_1_). In the camera coordinate system *O_C_-X_C_Y_C_Z_C_*, the coordinates of the end of welding torch conductive nozzle *P*_E_ are (*D*_0_, 0, −*H*_0_). where the sensor installation height *H*_0_ can be determined by calibration.

Thus, for the upper surface *S*_1_ of planar welded workpiece, the welding torch height *H* is the distance between point *P*_j_ and *P*_E_, which is:

(18) $$H=\;\frac{\;(A_{1}D_{0}+D_{1})}{\;C_{1}}-H_{0}$$

For the upper surface *S*_4_ of planar workpiece, the welding torch height *H* can also be calculated similarly. In practical application, the welding torch heights *H* relative to the two upper surfaces of *S*_1_ and *S*_4_, respectively, can be used alone or used with their mean value according to actual needs.

#### 4.4.3. Solution of Relative Posture Parameters of Welding Torch

During the arc welding process, the relative position parameters of welding torch affect the accuracy of the weld forming position and the relative posture parameters of welding torch (front and rear tilt angle *α*, left and right tilt angle *β*) affect the shape of weld pool and its fluidity, then will affect the quality of weld formation, including penetration, width and reinforcement. The values of relative posture parameters of welding torch are closely related to the absolute spatial posture of welding groove.

1. Front and rear tilt angle α of the welding torch

The bottom plane *S*_7_ is parallel to the upper surface of planar workpiece. Thus, the relationship between the direction vector of the line *P*_j0_*P*_j1_ in bottom plane *S*_7_ and the direction vector ***tor*** of the axis of welding torch can be used to characterize the front and rear tilt angle *α* of welding torch relative to the welding groove.

The direction vector of the line *P*_j0_*P*_j1_ is: ***q*_1_** = (*D*_0_, 0, −*A*_7_*D*_7_/*C*_7_). Take the value of the direction vector of the central axis of welding torch as: ***tor*** = (0, 0, 1). Therefore, the front and rear tilt angle *α* of welding torch can be solved, as follows:

(19) $$\alpha =-\arcsin\frac{\mathrm{tor}\;•\;q_{1}}{\left|\mathrm{tor}\right|\left|q_{1}\right|}$$

Considering the front and rear tilt angle *α* is generally expressed as an acute angle during the actual welding process. A positive value of *α* indicates the welding torch tilts forward relatively along welding direction, and a negative value of *α* indicates the welding torch tilts backward relatively along welding direction.

2. Left and right tilt angle β of the welding torch

The relationship between the direction vector ***tor*** of welding torch and the vector ***q***_2_, which is simultaneously perpendicular to the normal vector ***m***_7_ and the vector ***q***_1_, can be used to characterize the left and right tilt angle *β* of welding torch relative to the welding groove. The vector ***q***_2_ is:

***q*_2_*= m*_7_ × *q*_1_
*=*** (−*A*_7_*D*_7_*B*_7_/*C*_7_, *A*_7_^2^*D*_7_/*C*_7_ + *C*_7_*D*_0_, −*B*_7_*D*_0_)

Therefore, the left and right tilt angle *β* of the welding torch is:

(20) $$\beta =-\arcsin\frac{\mathrm{tor}\;•\;q_{2}}{\left|\mathrm{tor}\right|\left|q_{2}\right|}$$

At the same, considering the left and right tilt angle *β* is generally expressed as an acute angle during the actual welding process. A positive value of *β* indicates the welding torch slants to the left relatively in the welding groove cross section, and a negative value of *β* indicates the welding torch slants to the right relatively in the welding groove cross section.

According to the above derived detection algorithm of welding groove sizes and relative position and posture parameters of welding torch, in the process of practical application of the designed vision sensor based on combined laser structured lights, there is no need to preset special or fixed relative posture between the welding torch (or the vision sensor) and the welding groove, that is, it can be arbitrary. The whole process only requires the modulated laser lines can be imaged completely, and the integrated detection of the welding groove sizes and the relative SPP parameters of the welding torch can be realized. At the same time, it can be seen that the derived detection algorithms have few application restrictions and do not depend on other devices. Except that the sensor installation parameters *H*_0_ and *D*_0_ (both can be obtained by calibration) are used in the solutions of the relative SPP parameters of the welding torch, the solutions of other parameters only depend on the internal parameters of the designed vision sensor, which effectively improve its detection applicability.

## 5. Experimental Verification and Discussion

**Figure 10:**
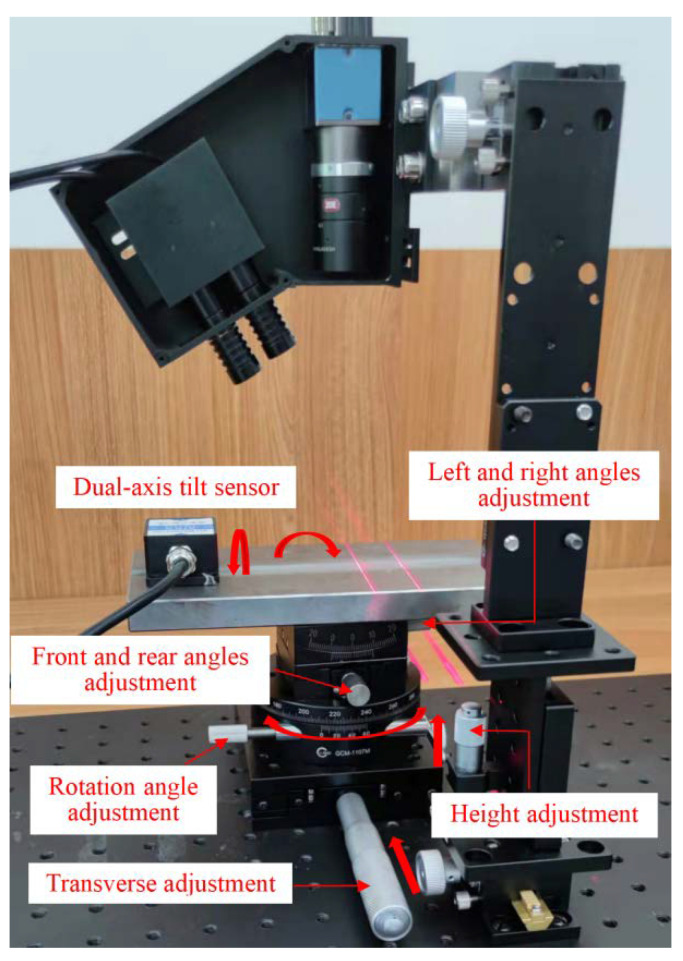
Parameters detection test platform based on designed vision sensor.

In order to verify the correctness of the internal parameter calibration method for the designed vision sensor proposed in this paper and the accuracy of calibration results, as well as the effectiveness of the above derived detection algorithms of welding groove sizes and the SPP parameters of the welding torch relative to the welding groove, some verification tests under three different conditions were carried out on the test platform shown in Figure 10. Considering the height of the welding torch during arc welding and the sensor installation height *H*_0_, the object distance of the camera is controlled at 140–160 mm in the verification tests, which can ensure that the magnification of the camera is similar in the verification tests, and the resolution of the camera is about 0.03–0.04 (mm/pixel) in the width direction and 0.07–0.10 (mm/pixel) in the depth direction in this case. These three tests were used to verify the detection accuracy of welding groove size parameters (groove depth *h*, groove width *b*_1_ and *b*_2_) and the spatial position parameters (*e*, *γ*, *H*) and posture parameters (*α*, *β*) of the welding torch relative to the welding groove separately.

T1: In test 1, symmetric and asymmetric V-grooves were used to verify the effectiveness and accuracy of detection algorithms of groove depth *h*, groove width *b*_1_ and *b*_2_, respectively. Table 3 shows the measured and detected values of welding groove sizes.

**Figure 11:**
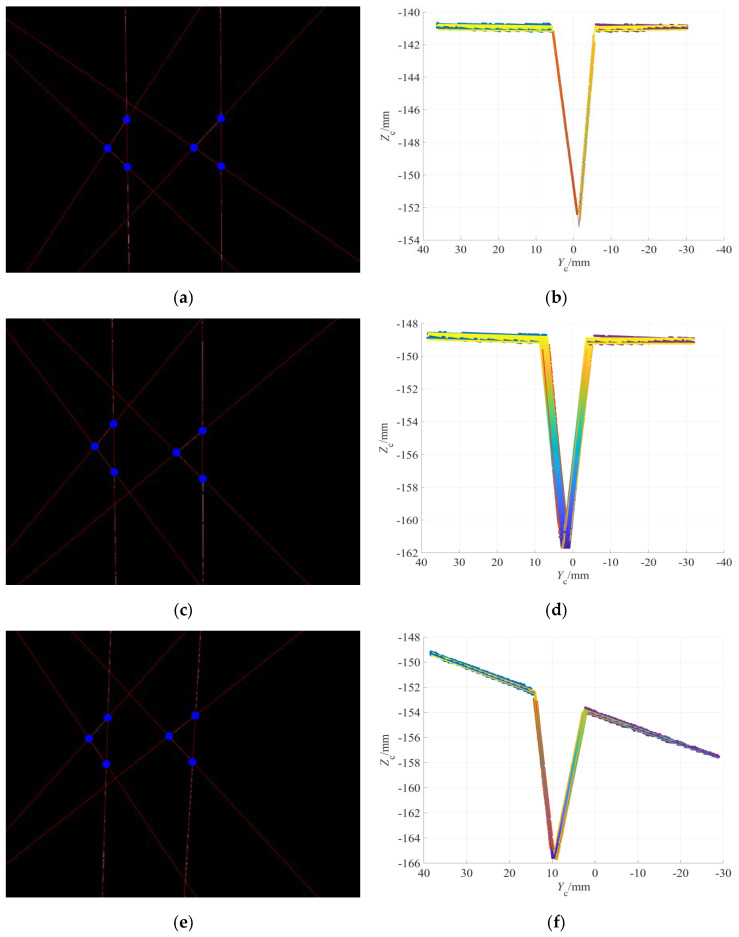
Detection test of welding groove sizes and relative position and posture parameters: (**a**) feature extraction for asymmetric V-groove image, (**b**) 3D reconstruction of asymmetric V-groove surfaces and its adjacent workpiece surfaces, (**c**) feature extraction for V-groove image with position deviation, (**d**) 3D reconstruction of V-groove surfaces and its adjacent workpiece surfaces with position deviation, (**e**) feature extraction for V-groove image with posture deviation and (**f**) 3D reconstruction of V-groove surfaces and its adjacent workpiece surfaces with posture deviation.

During the detection test of T1, on the premise of ensuring that the modulated laser lines are completely located in the field of vision of the camera, the workpiece was placed on the platform in any position and posture. The feature extraction diagram of the laser lines image modulated by typical V-groove and the 3D reconstruction diagram of the welding groove surfaces and its adjacent workpiece surfaces are shown in Figure 11a,b.

From Table 3, it can be seen that the maximum absolute error of welding groove size parameters between the detection value and measured value did not exceed 0.08 mm, the maximum relative error did not exceed 1% and the maximum repetition error did not exceed 0.04 mm. These detection results indicate the plane fitting method, which is used for the 3D reconstruction of welding groove surfaces and its adjacent workpiece surfaces, can greatly reduce the detection error caused by the deviation of feature point extraction. Meanwhile, the detection under any relative position and posture of workpiece can represent the variation of welding groove sizes caused by thermal deformation of the welded workpiece, so the detection method proposed can eliminate the impact of thermal deformation on the weld formation during the arc welding process to a certain extent. Therefore, the detection method has good detection accuracy, repeatability and adaptability.

T2: Since the central axis of the welding torch is coplanar with the optical axis of the CMOS camera and the central axes of two line laser emitters, and the central axis of the welding torch is parallel to the optical axis of the camera, the SPP of the camera relative to the welding groove can be used to characterize the SPP of the welding torch relative to the welding groove. In test 2, a symmetric V-groove was used in the detection test of the camera (characterizing welding torch) position parameters (*e*, *γ*, *H*_1_) relative to the welding groove. In order to facilitate the setting of position parameters and test verification, this test placed the planar workpiece with V-groove vertically under the camera, that is, the upper surface of planar workpiece was perpendicular to the optical axis of the camera. Under this condition, the camera height *H*_1_ can be expressed as: *H*_1_ = |*D*_1_/*C*_1_|, and the relationship between the torch height *H* and the camera height *H*_1_ is shown in Equation (14). As shown in Figure 10, the horizontal precision displacement platform was used to set the transverse deviation *e*, the rotary precision displacement platform was used to set the angular deviation *γ* and the vertical precision displacement platform was used to set the relative height *H*_1_ of the camera.

The set values of the precision displacement platform are taken as the measured values of the position deviation, and the position deviations obtained by vision sensing detection algorithms are taken as the detected values. Table 4 shows the measured and detected values of the position parameters of the camera (characterizing welding torch) relative to the welding groove. The feature extraction diagram of the laser lines image modulated by typical V-groove and the 3D reconstruction diagram of the welding groove surfaces and its adjacent workpiece surfaces are shown in Figure 11c,d.

From Table 4, it can be concluded that the maximum absolute errors of transverse deviation *e* and height *H*_1_ did not exceed 0.04 mm and the maximum absolute error of angular deviation *γ* did not exceed 0.1°. At the same time, the maximum relative errors of each parameter detection results were no more than 2%, which indicates the detection algorithms can be used for the accurate detection of the position of the welding torch relative to the welding groove and their real-time feedback adjustment.

T3: In test 3, a symmetric V-groove was used in the detection test of the camera (characterizing welding torch) posture parameters (*α*, *β*) relative to the welding groove. The dual-axis tilt sensor was placed on the upper surface of the planar workpiece to real-time display the posture parameter values of the planar workpiece.

In the test of T3, the front and rear tilt angle *α* and the left and right tilt angle *β* of the V-groove of the planar workpiece were respectively set by the two knobs of the biaxial angle adjusting platform in Figure 10. The reading values of the dual-axis tilt sensor were taken as the measured values of relative posture parameters and the calculated values obtained by vision detection algorithms were taken as the detected values of relative posture parameters. Table 5 shows the measured and detected values of the posture parameters of the camera (characterizing welding torch) relative to the welding groove. The feature extraction diagram of the laser lines image modulated by typical V-groove and the 3D reconstruction diagram of the welding groove surfaces and its adjacent workpiece surfaces are shown in Figure 11e,f.

From Table 5, it can be seen that the detection algorithms can also accurately detect the posture parameters of camera (characterizing welding torch) relative to the welding groove. The maximum absolute error of the detected values did not exceed 0.2° and the maximum relative error of the detected values were no more than 5%, which shows that this research work can fully meet the requirements of detection and control for relative posture of welding torch during the arc welding process.

Analyzing the above test results further, it is found that the main detection errors of test 2 and test 3 come from the fact that the initial posture of the camera (characterizing welding torch) relative to the welding groove is not an ideal vertical condition, which leads to the coupling among these parameters, and the measured values cannot fully represent the actual values of the parameters in the detection process of the relative position and posture parameters of the camera (characterizing welding torch).

In short, based on the processing of a single modulated laser lines image, the derived detection algorithms can effectively realize the integrated detection of the welding groove size parameters (*h*, *b*_1_, *b*_2_) as well as the SPP parameters (*e*, γ, *H* and *α*, *β*) of the welding torch relative to the welding groove. Their detection accuracy can fully meet the needs of actual welding production, which indicates the algorithms can provide strong support for on-line detection and feedback control of intelligent welding production.

## 6. Conclusions

There are three main research works accomplished in this paper. Some valuable conclusions could be obtained as follows:

(1) For the specially designed vision sensor based on combined laser structured lights, an integrated calibration method of vision sensor internal parameters is proposed, which uses only an ordinary planar checkerboard calibration board. The internal parameters (including camera internal parameters of *f*_x_, *f*_y_, *u*_0_, *v*_0_, *k*_1_, *k*_2_ and laser structured light plane equation parameters of *A_l_*_1_, *B_l_*_1_, *C_l_*_1_, *D_l_*_1_ and *A_l_*_2_, *B_l_*_2_, *C_l_*_2_, *D_l_*_2_ in the camera coordinate system) of the vision sensor can be integrated, calibrated effectively by the proposed calibration method. This reduces the requirement for high installation accuracy of two laser transmitters to a great extent, avoids the influence of non-parallel error in two laser structured light projection planes on detection results and eliminates the cumulative error in stepwise calibration. Thus, the proposed integrated calibration method improves the efficiency, accuracy and comprehensiveness of internal parameter calibration for a line structured light vision sensor and provides a good foundation for industrial application of the vision sensor.
(2) The derived high precision integrated detection algorithms for the V-groove size parameters (groove width *b*_1_, *b*_2_ and groove depth *h*) of the planar workpiece and the SPP parameters (including position parameters of *e,* *γ*, *H* and posture parameters of *α*, *β*) of the welding torch relative to the welding groove can be applied under any SPP of the welding torch (or vision sensor). Based on the 3D data of modulated laser lines obtained by the processing of the single modulated laser lines image, the algorithms reconstruct the 3D surfaces of V-groove surfaces and its adjacent surfaces of planar workpiece by data segmentation and plane fitting. This improves the utilization of modulated laser lines image data and reduces the interference of image processing error on the parameter detection.
(3) According to the proposed integrated calibration method and derived high precision integrated detection algorithms, some verification tests were carried out. The experimental results show that the derived integrated detection algorithms can be applied under any position and posture of the welding torch (or vision sensor) and has good detection accuracy and robustness, which improves the applicability of the vision sensor and the integration of detection algorithms. This work has important value for the application of the vision sensor in intelligent welding production.

## Figures and Tables

**Table 1:** Component selection and their main parameters of vision sensor.

Component Designations	Model and Main Parameters
Industrial camera	CMOS: MER2-503-23GM
	Resolution: 2448 × 2048
	Exposure mode: Global Shutter
	Exposure frequency: 23.5 fps
	Dimension of the sensor matrix: 2/3″
	Pixel size: 3.45 × 3.45 μm
Line laser emitter	Wavelength: 660 nm
	Lens: glass lens
	Size: Φ16 × 70 mm
	Power: 200 mW
	Focal length: adjustable
Camera lens	Model: Computar, M1228-MPW3
	Focal length: 12 mm
	Angle of view (D × H × V):49.3° × 40.3° × 30.8°
	Working distance: 100 mm~inf
	Maximum compatible target size: 2/3″
Filter	Filter wavelength: 660 ± 8 nm

**Table 2:** Calibration results of internal parameters for vision sensor.

Calibration Items	Calibration Parameters	Calibration Parameters Value
Internal parameters of the camera	*f*_x_	3544
	*f*_y_	3543
	*u*_0_	1239
	*v*_0_	1225
	*k*_1_	−0.0508
	*k*_2_	0.0738
Structured light plane equation parameters of two laser emitters	*A* *_l_*_1/_ *A* *_l_*_2_	0.8771/0.8748
	*B* *_l_*_1/_ *B* *_l_*_2_	0.1340/0.0044
	*C* *_l_*_1/_ *C* *_l_*_2_	0.4801/0.4844
	*D* *_l_*_1/_ *D* *_l_*_2_	51.63/71.57

**Table 3:** V-grooves size parameters detection of planar workpiece.

Groove Type	Size Parameters	Measured Value (mm)	Detected Value (mm)	Mean Detected Value (mm)	Standard Deviation (mm)	Absolute Error (mm)		Relative Error	
						Absolute Value	Maximum	Absolute Value	Maximum
Symmetric V-groove	*h*	13.065	13.070	13.093	0.033	0.005	0.074	0.04%	0.57%
			13.069			0.004		0.03%	
			13.139			0.074		0.57%	
	*b*_1_	5.976	5.980	5.981	0.009	0.004	0.016	0.07%	0.27%
			5.992			0.016		0.27%	
			5.972			0.004		0.07%	
	*b*_2_	6.022	6.002	6.021	0.021	0.020	0.029	0.33%	0.48%
			6.011			0.011		0.18%	
			6.051			0.029		0.48%	
Asymmetric V-groove	*h*	12.071	12.134	12.137	0.002	0.063	0.069	0.52%	0.57%
			12.137			0.066		0.55%	
			12.140			0.069		0.57%	
	*b*_1_	6.996	6.990	7.000	0.010	0.006	0.017	0.09%	0.24%
			6.996			0.00		0.00%	
			7.013			0.017		0.24%	
	*b*_2_	4.425	4.465	4.454	0.011	0.040	0.040	0.90%	0.90%
			4.458			0.033		0.75%	
			4.440			0.015		0.34%	

**Table 4:** Position parameters detection of camera (characterizing welding torch) relative to welding groove.

Position Parameters	Measured Value	Detected Value	Absolute Error		Relative Error	
			Absolute Value	Maximum	Absolute Value	Maximum
*e*/mm	3	2.996	0.004	0.006	0.13%	0.13%
	5	5.006	0.006		0.12%	
	8	8.004	0.004		0.05%	
*γ*/°	1.6	1.613	0.013	0.085	0.81%	1.77%
	3.2	3.158	0.042		1.31%	
	4.8	4.715	0.085		1.77%	
*H*_1_/mm	143	143.011	0.011	0.040	0.01%	0.03%
	148	147.960	0.040		0.03%	
	153	153.009	0.009		0.01%	

**Table 5:** Posture parameters detection of camera (characterizing welding torch) relative to welding groove.

Posture Parameters	Measured Value	Detected Value	Absolute Error		Relative Error	
			Absolute Value	Maximum	Absolute Value	Maximum
*α*/°	−4.94	−4.862	0.078	0.124	1.58%	4.40%
	−2.56	−2.549	0.011		0.43%	
	2.82	2.696	0.124		4.40%	
*β*/°	−10.06	−10.131	0.071	0.071	0.71%	2.75%
	2.18	2.240	0.060		2.75%	
	4.36	4.372	0.012		0.28%	

## Data Availability

Not applicable.
